# Commissioning of the NPDGamma Detector Array: Counting Statistics in Current Mode Operation and Parity Violation in the Capture of Cold Neutrons on *B*_4_*C* and ^27^*Al*

**DOI:** 10.6028/jres.110.027

**Published:** 2005-06-01

**Authors:** M. T. Gericke, J. D. Bowman, R. D. Carlini, T. E. Chupp, K. P. Coulter, M. Dabaghyan, D. Desai, S. J. Freedman, T. R. Gentile, R. C. Gillis, G. L. Greene, F. W. Hersman, T. Ino, S. Ishimoto, G. L. Jones, B. Lauss, M. B. Leuschner, B. Losowski, R. Mahurin, Y. Masuda, G. S. Mitchell, S. Muto, H. Nann, S. A. Page, S. I. Penttila, W. D. Ramsay, S. Santra, P.-N. Seo, E. I. Sharapov, T. B. Smith, W. M. Snow, W. S. Wilburn, V. Yuan, H. Zhu

**Affiliations:** Los Alamos National Laboratory, Los Alamos, New Mexico 87545, USA; Indiana University, Bloomington, Indiana 47405, USA; Indiana University, Bloomington, Indiana 47405, USA; Thomas Jefferson National Accelerator Facility, Newport News VA 23606, USA; University of Michigan, Ann Arbor, MI 48104, USA; University of New Hampshire, Durham, NH 03824, USA; University of Tennessee, Knoxville, TN 37996, USA; University of California, Berkeley, CA 94720-7300, USA; National Institute of Standards and Technology, Gaithersburg, MD 20899-0001, USA; University of Manitoba, Winnipeg, Manitoba R3T 2N2, Canada; Oak Ridge National Laboratory, Oak Ridge, TN 37831, USA; niversity of Tennessee, Knoxville, TN 37996, USA; University of New Hampshire, Durham, NH 03824, USA; High Energy Accelerator Research Organization (KEK), Tukubash-shi, 305-0801, Japan; Hamilton College, Clinton, NY 13323, USA; University of California, Berkeley, CA 94720-7300, USA; Indiana University, Bloomington, Indiana 47405, USA; University of Tennessee, Knoxville, TN 37996, USA; High Energy Accelerator Research Organization (KEK), Tukubash-shi, 305-0801, Japan; Los Alamos National Laboratory, Los Alamos, New Mexico 87545, USA; High Energy Accelerator Research Organization (KEK), Tukubash-shi, 305-0801, Japan; Indiana University, Bloomington, Indiana 47405, USA; University of Manitoba, Winnipeg, Manitoba R3T 2N2, Canada; Los Alamos National Laboratory, Los Alamos, New Mexico 87545, USA; TRIUMF, 4004 Wesbrook Mall, Vancouver, British Columbia V6T 2A3, Canada; Indiana University, Bloomington, Indiana 47405, USA; Los Alamos National Laboratory, Los Alamos, New Mexico 87545, USA; Joint Institute for Nuclear Research, Dubna, Russia; University of Dayton, Dayton, OH 45469, USA; Indiana University, Bloomington, Indiana 47405, USA; Los Alamos National Laboratory, Los Alamos, New Mexico 87545, USA; University of New Hampshire, Durham, NH 03824, USA

**Keywords:** CsI, current mode, detector, gamma, neutron capture, parity violation

## Abstract

The NPDGamma γ-ray detector has been built to measure, with high accuracy, the size of the small parity-violating asymmetry in the angular distribution of gamma rays from the capture of polarized cold neutrons by protons. The high cold neutron flux at the Los Alamos Neutron Scattering Center (LANSCE) spallation neutron source and control of systematic errors require the use of current mode detection with vacuum photodiodes and low-noise solid-state preamplifiers. We show that the detector array operates at counting statistics and that the asymmetries due to B_4_C and ^27^Al are zero to with- in 2 × 10^−6^ and 7 × 10^−7^, respectively. Boron and aluminum are used throughout the experiment. The results presented here are preliminary.

## 1. Introduction

The NPDGamma experiment is under commissioning at the Los Alamos Neutron Scattering Center (LANSCE). It is the first experiment that was designed for the new pulsed high flux cold beam line, flight path 12, at LANSCE. NPDGamma will determine the very small weak pion-nucleon coupling constant *f*_π_ in the nucleon-nucleon interaction [1–5]. This coupling constant is directly proportional to the parity-violating up-down asymmetry *A*_γ_ in the angular distribution of 2.2 MeV gamma rays with respect to the neutron spin direction in the reaction $\vec{n}\;+\;p\to d\;+\;\gamma$. The asymmetry has a predicted size of 5 × 10^−8^ and our goal is to measure it to 10%.

The small size of the asymmetry and the high proposed measurement precision impose heavy requirements on the performance of the beam line and apparatus. It is necessary to achieve high counting statistics while at the same time suppressing any systematic errors below the statistical limit. The detector array was designed to satisfy these requirements [6,7].

The experiment uses an intense, pulsed (20 Hz), cold neutron beam at LANSCE [8]. The beam is transversely polarized by transmission through a polarized ^3^He cell. Three ^3^He ion chambers are used to monitor beam intensity and measure beam polarization through transmission ratios. A radio frequency spin flipper is used to reverse the neutron spin direction on a pulse by pulse basis. For the production experiment the neutrons will capture in a 21 L liquid para-hydrogen target. The gamma rays from the capture are detected by an array of 48 CsI(Tl) detectors. The entire apparatus is located in a homogeneous 1 mT magnetic field to maintain the neutron spin downstream of the polarizer. A detailed introduction to the experiment is given in [1].

During commissioning the radiative neutron capture on various target materials was investigated to look for any false asymmetry which may enter as a systematic effect while taking data with the hydrogen target. The measurements concentrated on materials that can be found within the experimental apparatus and which are interacting with the neutrons. The targets used included Al, Cu, In, ^6^Li, and B_4_C.

Boron is used throughout the experiment, for neutron shielding and to collimate the beam. Aluminum is used in most of the equipment and the beam encounters several millimeter of it, primarily in the windows of the hydrogen target. It is therefore necessary to establish the size of the γ-asymmetry due to neutron capture on both of these elements. A preliminary result on the aluminum asymmetry is presented in [1]. The preliminary asymmetry result for the B_4_C data taken so far is presented here. Further analysis on all targets is currently underway.

To establish that the array operates at counting statistics, it is desirable to conduct a neutron capture experiment in which a single neutron capture produces at most one outgoing gamma ray with a known energy. The reaction ^10^B(n,α)^7^Li produces an excited ^7^Li nucleus with a branching ratio of 94 %. The excited nucleus decays, emitting a 0.48 MeV gamma ray, 100 % of the time. As a result, the width in the data taken with a B_4_C target can be compared to the width expected from neutron counting statistics.

Based on LANSCE FP12 beam brightness measurements and Monte Carlo calculations [8], the predicted average gamma rate into a single detector is 20 MHz. At that rate, pulse counting is not possible, given the long decay time of the scintillation light pulses in CsI. The detectors are therefore operated in current mode. The scintillation light from the CsI detectors is converted to current signals using vacuum photodiodes (VPD), and the photocurrents are converted to voltages and amplified by solid-state electronics [7].

**Fig. 1:**
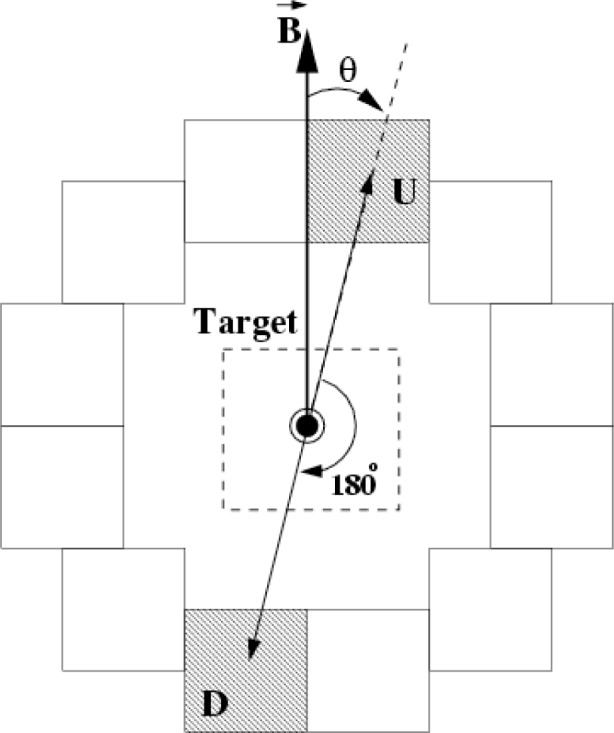
A ring of detectors and one up-down pair, as seen in beam direction. $\vec{B}$ is the magnetic holding field defining the direction of the neutron polarization.

The 48 detectors are grouped in rings of 12 detectors each (Fig. 1) and arranged in a cylindrical pattern around the neutron capture target used. The detector signals are sampled by 16 bit analog-to-digital converters (ADCs) and integrated over a 0.4 ms period, defining the time bin width at which the detector signals are stored. For each neutron pulse, a total of 100 time bins per detector is stored, followed by a 10 ms break before the next frame of neutrons arrives. This 50 ms period defines a *macro pulse* of data.

The remainder of this paper gives a short description of the counting statistics and asymmetry tests performed and the relevant preliminary results obtained.

## 2. Achieving Counting Statistics in Current Mode Operation

In current mode detection, counting statistics appears as the RMS width in the sample distribution, due to the fluctuation in the number of electrons produced at the photocathode of the VPD, as a result of the quasi instantaneous amount of energy deposited in the CsI crystal.

During beam on measurements, the shot noise RMS width is given by [9]

$$\sigma I_{\text{shot}}=\sqrt{2qI}\sqrt{f_{B}},$$ (1)

where *q* is the amount of charge created by the photocathode per detected gamma-ray, *I* is the average photocurrent per detector and *f*_B_ is the sampling bandwidth, set by the 0.4 ms time bin width.

The boron target used in this measurement consisted of a 1 cm thick 15 cm by 15 cm sheet of sintered B_4_C located in the center of the detector array. The B_4_C target is black for cold neutrons. The beam was collimated down to a 10 cm diameter cross-section, so that the target completely covered the beam. Three consecutive measurements were made. Three 500 s long pedestal runs, without beam, one 500 s data run with beam incident on the target and one 500 s background run with beam but without the target installed.

Because of the single gamma released in the ^10^B(n, α)^7^Li capture reaction and Eq. (1) a direct comparison can be made between the RMS width expected from counting statistics and the RMS width seen in the data stream. Several factors contribute to the overall width seen in the data stream. Contributions from electronic noise and signals from activation decay in the various materials are measured during beam-off, pedestal runs. Contributions from radiative capture and scattering on materials other than the intended target are measured by performing background runs. Both of these contributions can be removed by subtracting their RMS widths in quadrature from the overall width. In addition, any fluctuations in the magnitude of the signal over the course of the measurement will also contribute to the width. To remove those contributions, the data and background runs were normalized to the beam current and separate variances were calculated for each group of 20 *macro pulses* and then averaged. For all of these calculations a single time bin, corresponding to the peak signal (*E* = 14 meV), was used in each macro pulse.

**Fig. 2:**
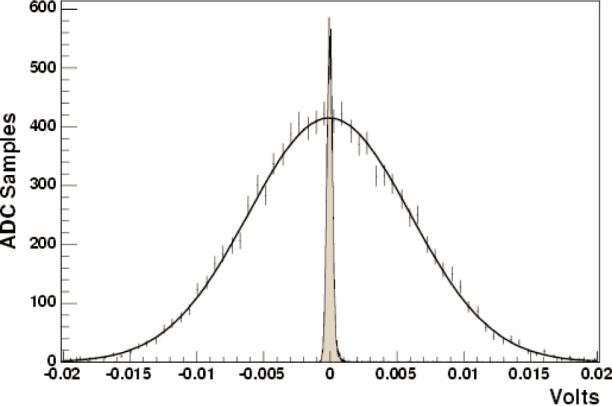
Counting statistics analysis results for a typical detector. The RMS width due to counting statistics is compared to the width seen from pedestal runs (electronic noise). The fit to the beam on, target in data histogram shows an RMS width of 6.1 mV ± 0.04 mV.

Figure 2 shows the RMS width for a typical detector, as seen at the preamplifier output. For this detector, using Eq. (1), the measured values for *q* and *I* and the gain of the preamplifier circuit, the RMS width expected at the preamplifier output is 5.7 mV ± 0.3 mV. The error on the expected width is dominated by the accuracy to which we know the efficiency (number of photoelectrons per MeV) of the detector. Additional contributions to the width, that are not currently accounted for, include fluctuations in the amount of energy a gamma-ray actually deposits in the detector (currently, it is assumed that each photon deposits its full energy) and background due to neutrons that are scattered off the target and subsequently capture in other surrounding materials. An effort to carefully model these effects, using Monte-Carlo methods is currently underway.

Using the known proton current and an appropriate Monte-Carlo model to simulate the transmission of the neutrons through the guide and the experiment, the number of neutrons per pulse expected to capture on the B_4_C target, for the peak energy, is about 8 × 10^6^ ms^−1^. Considering the gamma attenuation in the target as well as the solid angle for the detector, the number of photons into a detector, per time bin is 3 × 10^4^, so that the relative width is $\frac{1}{\sqrt{N}}≃0.6\%$. Using the corresponding voltage, one expects to see out of the preamplifier, the RMS width expected due to neutron counting statistics is ≃5 mV±1 mV. The error is due to the accuracy to which we know the detector efficiency (≈5 %), the estimate on the accuracy in the number of neutrons capturing in the target (≈5 %), but mostly due to an error estimate on the number of gamma rays that will deposit all of their energy in the detector (≈20 %).

## 3 The Parity Violating γ-asymmetry in ^10^B(n, γ)^11^B and ^27^Al(n, γ)^28^Al

Due to the 1 mT holding field surrounding the experimental apparatus, the neutrons are polarized vertically after leaving the ^3^He spin filter. While taking hydrogen data, the parity violating asymmetry in n-p capture is therefore seen in a difference of the number of γ rays going up and down. The cross section is proportional to 1 *+A_γ_* cos*θ*, where *θ* is the angle between the neutron polarization and the momentum of the emitted photon. Accordingly, when investigating possible *false* asymmetries, i.e., those not due to n-p capture in the hydrogen target, we look for them in the up-down direction with respect to the neutron polarization axis.

The geometry of the B_4_C target was described earlier. The aluminum target consisted of 30 sheets of aluminum, each of which was ≈1.6 mm thick. Each sheet was separated by 5 mm to reduce gamma attenuation in the target. The total target thickness was 5 cm and its cross-section was 9 cm by 9 cm. The target was located in the center of the array.

During the first phase of commissioning, approximately 1.5 hours of B_4_C data were taken and the results show a zero asymmetry at the 2 × 10^−6^ level of accuracy. The aluminum asymmetry was measured to be zero at the 0.73 × 10^−6^ level using about 40 h of data.

In calculating the asymmetry for the entire period, one asymmetry was calculated for each detector pair and time bin and over any valid sequence of eight macro pulses with the correct neutron spin state pattern. A so-called valid eight step sequence of spin states is defined as (↑↓↓↑↓↑↑↓). Using this pattern suppresses first and second order gain drifts within the sequence. Some eight step sequences may become invalid due to missing pulses in the DAQ.

A pair of detectors is defined as shown in Fig. 1. If we let (*U*_↑_, *U_↓_* or *D_↑_, D↓*) denote the sum of all four signals with the corresponding spin states in a given spin sequence, then the detector pair asymmetry for a given time bin is calculated from

$$A_{\text{raw}}\left(t_{i}\right)=\frac{U_{↑}-D_{↑}-U_{↓}+D_{↓}}{U_{↑}+D_{↑}+U_{↓}+D_{↓}}.$$ (2)

This method of calculating the asymmetry allows for automatic subtraction of pedestals and any additive background. Signal fluctuations that are not correlated with the switching of the neutron polarization direction will average out and don’t contribute to any asymmetry. Possible false asymmetries due to electronic pickup and possible magnetic field induced gain changes in the detector VPDs have previously been measured and are consistent with zero to within 5 × 10^−9^ [6].

The asymmetry was then divided by the neutron polarization and the cosine of the corresponding detector pair angle. For each detector pair the resulting time bin physics asymmetries within a sequence were combined by calculating an error weighted average over the time of flight selected for analysis (13 ms through 34 ms). A 48 % ^3^He polarization was calculated from the monitor ratios and was seen to be stable over the analyzed runs.

**Fig. 3:**
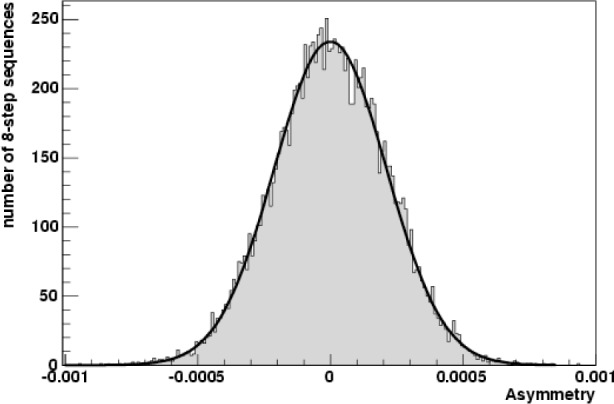
Up-Down gamma-ray asymmetry for ≈1.5 h of ^10^B data. From the fit, the asymmetry is (−0.2 ± 1.9) × 10^−6^.

The spin-flipper efficiency was taken to be 100 % over the analyzed time of flight range. The resulting physics asymmetry for each valid eight step sequence was then histogrammed to produce the total asymmetry. The results for the asymmetry are shown in Fig. 3.

**Fig. 4:**
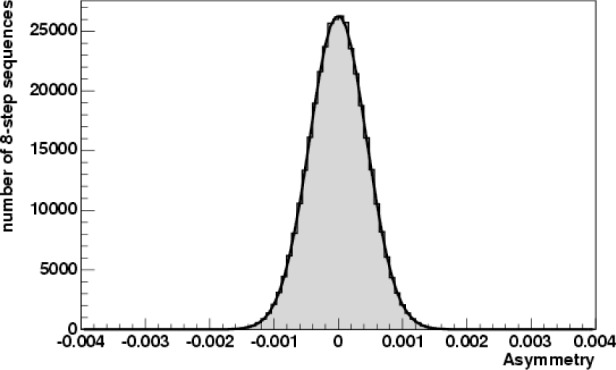
Up-Down gamma-ray asymmetry for ≈40 h of ^27^Al data. From the fit, the asymmetry is (−0.95 ± 0.73) × 10^−6^.

The asymmetry on aluminum was calculated using the same method. The results are shown in Fig. 4.

## 4. Conclusion

The NPDGamma experiment successfully completed a commissioning run in April 2004. The CsI detector array was demonstrated to be sufficiently low noise to allow operation at counting statistics. The array was used to perform measurements of asymmetries in neutron capture in gamma-ray emission following capture of ^10^B and ^27^Al. Given the thickness of the targets used and the amount of actual material that is estimated to interact with the neutrons, about 10 % of the signal seen with the hydrogen target will be due to aluminum while about 1 % comes from boron. The amount of aluminum data analyzed corresponds to about 1/3 of the data taken during the commissioning run. Taking these factors into account and using all collected data, we will be able to show that neither material is going to contribute a false asymmetry in the background signal of the NPDGamma apparatus. The CsI detector array is fully commissioned and prepared for production data taking with the hydrogen target.
